# Widespread Separation of the Polypyrimidine Tract From 3′ AG by G Tracts in Association With Alternative Exons in Metazoa and Plants

**DOI:** 10.3389/fgene.2018.00741

**Published:** 2019-01-14

**Authors:** Hai Nguyen, Jiuyong Xie

**Affiliations:** ^1^Department of Physiology and Pathophysiology, Max Rady College of Medicine, Rady Faculty of Health Sciences, University of Manitoba, Winnipeg, MB, Canada; ^2^Department of Applied Computer Sciences, University of Winnipeg, Winnipeg, MB, Canada

**Keywords:** 3′ splice site, intron, branch point, species, cancer

## Abstract

At the end of introns, the polypyrimidine tract (Py) is often close to the 3′ AG in a consensus (Y)_20_NCAGgt in humans. Interestingly, we have found that they could also be separated by purine-rich elements including G tracts in thousands of human genes. These regulatory elements between the Py and 3′ AG (REPA) mainly regulate alternative 3′ splice sites (3′ SS) and intron retention. Here we show their widespread distribution and special properties across kingdoms. The purine-rich 3′ SS are found in up to about 60% of the introns among more than 1,000 species/lineages by whole genome analysis, and up to 18% of these introns contain the REPA G-tracts (REPAG) in about 0.6 million of 3′ SS in total. In particular, they are significantly enriched over their 3′ SS and genome backgrounds in metazoa and plants, and highly associated with alternative splicing of genes in diverse functional clusters. Cryptic splice sites harboring such G- and the other purine-triplets tend to be enriched (2–9 folds over the disrupted canonical 3′ SS) and aberrantly used in cancer patients carrying mutations of the SF3B1 or U2AF35, factors critical for branch point (BP) or 3′ AG recognition, respectively. Moreover, the REPAGs are significantly associated with reduced occurrences of BP motifs between the −24 and −4 positions, in particular absent between the −7 and −5 positions in several model organisms examined. The more distant BPs are associated with increased occurrences of alternative splicing in humans and zebrafish. The REPAGs appear to have evolved in a species- or phylum-specific way. Thus, there is widespread separation of the Py and 3′ AG by REPAGs that have evolved differentially. This special 3′ SS arrangement likely contributes to the generation of diverse transcript or protein isoforms in biological functions or diseases through alternative or aberrant splicing.

## Introduction

Splice sites demarcate the boundaries between introns and exons for proper splicing of precursor RNA transcripts. Their sequences are constrained by a consensus but could be highly diverse among hundreds of species (Nguyen et al., 2018). The diversity and flexibility may contribute to alternative pre-mRNA splicing as well as to the mutation effect in many diseases (Roca et al., 2012; Feng and Xie, 2013; Sohail and Xie, 2015a,b). The majority of 3′ splice sites (3′ SS) are comprised of the branch point (BP), polypyrimidine tract and 3′ AG dinucleotides with a consensus (Y)_20_NCAGgt in humans based on the whole genome data (Nguyen et al., 2018). These motifs are recognized by splicing complexes/factors U2 snRNP, U2AF65 and U2AF35, respectively (Black et al., 1985; Kramer, 1987; Singh et al., 1995; Merendino et al., 1999; Wu et al., 1999; Zorio and Blumenthal, 1999). Mutations of SF3B1 of the U2 snRNP complex and U2AF35 cause aberrant 3′ splice site usage in leukemia, melanoma, breast and lung cancers (Przychodzen et al., 2013; Brooks et al., 2014; Darman et al., 2015; Alsafadi et al., 2016; Kesarwani et al., 2017).

Unlike the 3′ SS consensus sequence where the Py and 3′ AG are adjacent to each other separated by only two nucleotides ‘NC’, we have found a group of intron ends where they are separated further apart by RNA elements (Xie et al., 2005; Sohail et al., 2014; Sohail and Xie, 2015a,b). The first identified is the CA-rich, Ca^++^/calmodulin-dependent protein kinase IV (CaMKIV)-responsive RNA element (CaRRE) (Xie and Black, 2001; Xie et al., 2005), which inhibits 3′ SS usage through hnRNP L/LL (Yu et al., 2009; Liu et al., 2012). We have further identified purine-rich including GGG elements within this region (Sohail et al., 2014). The G tracts inhibit U2AF65 binding and 3′ SS usage through hnRNP H/F contributing to the emergence of novel alternative exons (Sohail et al., 2014; Sohail and Xie, 2015b). Together, we call these regulatory elements between the Py and 3′ AG (REPA) (Sohail et al., 2014). The REPA G tract (REPAG)-containing human genes are significantly enriched for cancer-related functions (Sohail et al., 2014; Sohail and Xie, 2015b).

Analysis of individual 3′ SS indicates that the human REPAGs were ‘inserted’ between the Py and 3′ AG mostly in the ancestors of mammalian genes during evolution (Sohail et al., 2014; Sohail and Xie, 2015b); however, their genome-wide prevalence and relationship to the upstream BP and alternative splicing among different species remain unclear. In this report, we examine their distribution in individual 3′ SS among > 1,000 Ensembl-annotated species/lineages, association with alternative splicing, their diverse host genes including those with aberrant splice sites in cancer, and association with distant BP motifs.

## Results

### Distribution of REPAG-Harboring 3′ Splice Sites Among More Than 1,000 Eukaryotic Species/Lineages

**FIGURE 1:**
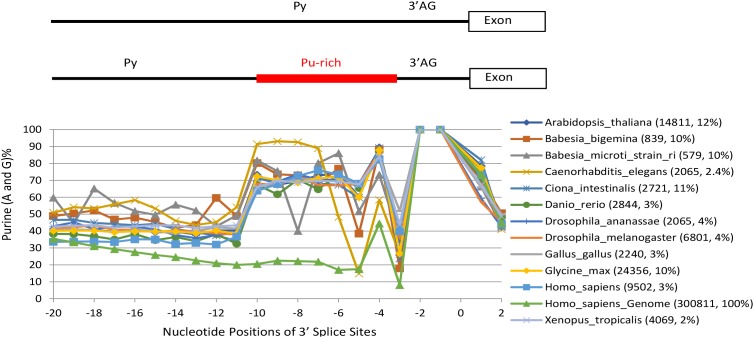
Percent distribution of purines (Pu) in the Pu-rich 3′ SS between the Py and 3′ AG (–10 and –3) of a class of introns in genomes representatives of 1,175 species/lineages. For convenience to trace the position-to-position changes in each species, their marked points of the purine nucleotide-percentages of each position are shown in straight line-linked curves. The extreme points such as those at –8 and –5 positions in the *Babesia microti* and *Glycine max* are due to constraints at these positions for Ts as reported by *Hai et al., Gene, 2018*. The distinct curve at the bottom between –20 and –3 is a control using the whole human genome. Bracketed next to the species names are the numbers of purine-rich 3′ SS and their percentages of all 3′ SS in the whole genome of each species. Position ‘0’ marks the intron-exon junction. Please see also Supplementary Table S1 for the purine-rich 3′ SS matrices of all the species/lineages, and Supplementary Data Files S1, S2 for all the GGG-harboring 3′ SS sequences.

**FIGURE 2:**
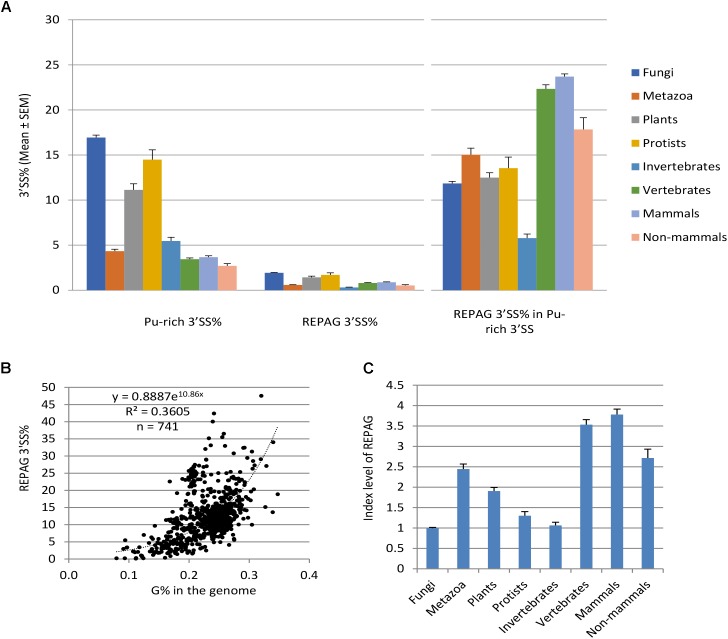
Distribution and enrichment of the REPAG motifs among different species/divisions. **(A)** Percent distribution (Mean ± SEM) of the purine-rich 3′ SS and REPAG 3′ SS in the genomes of the four eukaryotic divisions, invertebrates and vertebrates of metazoa, mammals and non-mammals of vertebrates. Also shown is the percent distribution of REPAG 3′ SS in the purine-rich 3′ SS (Right panel). *n* = 434, 161, 52, 94, 71, 90, 69 and 21 species, for the 8 groups, respectively. Data from 741 unique species with > 100 purine-rich 3′ SS are included. **(B)** Plot of the REPAG 3′ SS% of the purine-rich 3′ SS versus the guanine nucleotide G% of the genomes of the 741 species. The equation inside the graph is for the fitted trendline. **(C)** REPAG enrichment index levels of the 8 groups (Mean ± SEM) after normalization to the purine-rich 3′ SS% and genome G% of each species. For this, the REPAG 3′ SS% in **(B)** was divided by the y values of the trendline corresponding to the G% of each species. The resulting mean value of fungal species is taken as 1.0 for comparison. Please see also Supplementary Table S2 for the REPAG 3′ SS matrices and Supplementary Table S3 for the enrichment of REPAG 3′ SS versus the purine-rich 3′ SS and genome G% of all species/lineages, and Supplementary Data Files S1, S2 for all the REPAG 3′ SS sequences.

We first calculated and identified 4,410,921 annotated purine-rich 3′ SS (≥ 5 purines between the −10 and −3 positions) of 1,175 eukaryotic species/lineages in the Ensembl releases R38/R91 (Figure 1, also Supplementary Table S1 for matrices of each species), as in our previous reports (Sohail et al., 2014; Nguyen et al., 2018). The purine enrichment contrasts the Py-rich content of the average 3′ SS matrices of the human or other genomes (Nguyen et al., 2018). The highest percentage of purine-rich 3′ SS is 61% in the genome of fungus *Edhazardia aedis*, which is A/T-rich (38% each). Even in species that are highly constrained at certain positions for Ts within this region (Hollins et al., 2005; Nguyen et al., 2018), such as the T_−5_ and T_−8_ in *C. elegans* and *B. microti*, respectively, and to a less extent the T_−5_ in *B. bigemina*, we still identified hundreds or thousands of purine-rich 3′ SS (Figure 1). The purine-rich 3′ SS are most abundant in fungi, comprising about 17% of all of the 3′ SS among the 434 species (Figure 2A, mean ± SEM), followed by protists and plants, 14 and 11%, respectively, and the least (4%) in metazoans (particularly vertebrates, 3.5%, and mammals, < 3%).

We identified 588,678 REPA G tracts (REPAG), the most prominent of the 3′ SS purine-rich motifs (Sohail et al., 2014; Sohail and Xie, 2015b), between the −15 and −3 positions among 1,031 species/lineages (Figure 2A, Supplementary Tables S2, S3, and Supplementary Data Files S1, S2 for matrices and sequences), about 13% of all of the purine-rich 3′ SS identified. Their percent distribution among the eukaryotic groups is similar to the purine-rich 3′ SS except that vertebrates (mammals in particular) have higher level than invertebrates (Figure 2A). However, relative to the purine-rich 3′ SS, REPAG 3′ SS are most enriched (15%) in metazoans and the least (12%) in fungi among the four divisions. Of the metazoans, vertebrates have the highest level, ∼22% (24% for mammals, 18% non-mammals), consistent with a previous genome-wide observation of G tracts (Yeo et al., 2004), while invertebrates have only ∼6%. Upon further normalization to the G% in the genomes (Figure 2B), the metazoa (vertebrates and mammals in particular) and plants are most enriched of the REPAGs (Figure 2C). Therefore, separation of the Py and 3′ AG by REPAGs is widespread and most enriched in metazoa and plants.

### The REPAG 3′ SS Are Highly Associated With Immediate Downstream Alternative Exons of Metazoan and Plant Genes of Diverse Functions

The REPAGs regulate the alternative splicing of a large group of exons in human genes involved in cancer and cell cycle (Sohail et al., 2014; Sohail and Xie, 2015b). To determine if they are also associated with alternative exons among the other species, we examined all exons immediately downstream of the REPAGs for their constitutive or non-constitutive usage in the Ensembl database. Exons with both coordinates present in all transcripts in the database are taken as constitutive ones; or else, non-constitutive or alternative exons.

**FIGURE 3:**
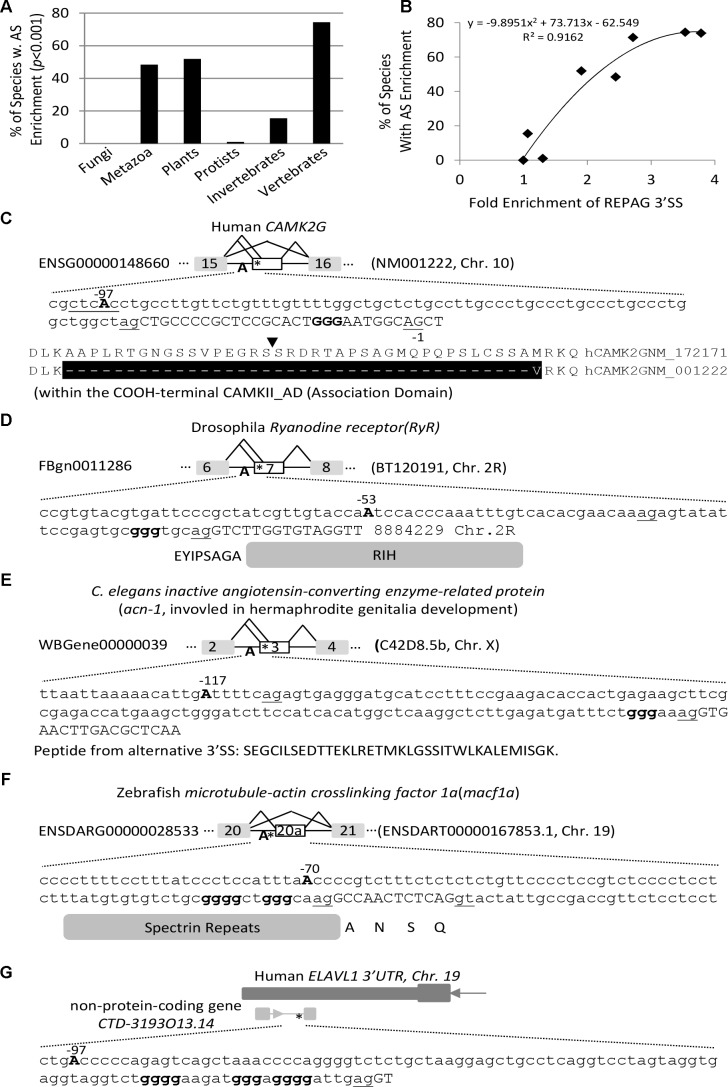
Enrichment of alternative exons downstream of the REPAG 3′ SS among 198 species/lineages. **(A)** Percentages of species that show highly significant enrichment (*p* < 0.001) of alternative exons downstream of the REPAG 3′ SS in each division/group (*n* = 123 unique species in total). **(B)** Correlation between the species percentages and the fold enrichment of REPAG 3′ SS (over fungal species) in each division/group. **(C–G)** Representative examples of 3′ SS and alternative exons of protein-coding and non-protein-coding genes of several species. Uppercases are exon and lowercases intron nucleotides. ^∗^location of GGG. The 3′ AG is underlined. The branch point (BP) Adenosine A is in bold and uppercase, and position is relative to the intron end G nucleotide (–1).

Compared to all exons in the transcriptome of each species, exons immediately downstream of the REPAGs are significantly enriched with non-constitutive ones in 198 species/lineages (*p* < 0.05, Figure 3 and Supplementary Table S4), and highly significant (*p* < 0.001) in 123 lineages of 106 unique species. Of the latter, 73% are metazoan, 25% plant and less than 1% protist species; of the metazoans, 89% are vertebrates and 11% are invertebrates. The 106 species represent 48% of metazoan (73% of vertebrates in particular, with 74% of mammals and 71% of non-mammals), 52% of plant and 1% of protist species that have the REPAG 3′ SS (Figure 3A). Of the vertebrates, the primates (night monkey *Aotus Nancymaae* and chimpanzee *Pan troglodytes*) are among the top ones by enrichment folds and *p*-values (Table 1). Of the plants, the common wheat *Triticum aestivum* and cotton *Gossypium raimondii* are among the top ones. There are no fungi among the 106 species. Overall, the percentages of such alternative splicing-enriched species in each division or group is well correlated with the enrichment folds of the REPAG 3′ SS (Figure 3B, *R*^2^ = 0.9162). Therefore, the REPAG 3′ SS are associated with alternative exons in more than a hundred species of mainly metazoans (vertebrates in particular) and plants.

**Table 1:** Top 10 species most significantly enriched of alternative exons downstream of REPAG 3′ SS in metazoa and plants, ranked by *p*-value.

Division	Genus	Species	a	b	c	d	*p*-Value
Metazoa	*Aotus*	*nancymaae*	62.3	2695	86.9	1.39	5.40E-181
	*Pan*	*troglodytes*	65.8	2518	90.2	1.37	1.47E-180
	*Papio*	*anubis*	62.8	2484	87.8	1.40	2.41E-175
	*Callithrix*	*jacchus*	65.2	1975	91.1	1.40	4.48E-161
	*Rhinopithecus*	*bieti*	65.9	2662	88.4	1.34	4.40E-159
	*Macaca*	*mulatto*	71.1	2756	91.6	1.29	1.00E-157
	*Cercocebus*	*atys*	64.2	2376	87.9	1.37	8.78E-154
	*Rhinopithecus*	*roxellana*	64.0	2185	88.3	1.38	4.63E-148
	*Macaca*	*nemestrina*	64.4	2394	87.3	1.36	5.57E-144
	*Mandrillus*	*leucophaeus*	66.4	2230	89.2	1.34	3.25E-140
Plants	*Triticum*	*aestivum*	70.3	5908	83.9	1.19	4.47E-131
	*Gossypium*	*raimondii*	63.4	2110	79.1	1.25	7.14E-56
	*Oryza*	*rufipogon*	76.9	2317	88.3	1.15	9.54E-46
	*Glycine*	*max*	68.2	2558	80.2	1.18	4.10E-43
	*Oryza*	*glumaepatula*	76.1	2287	87.5	1.15	4.12E-43
	*Oryza*	*nivara*	75.3	2267	86.8	1.15	9.79E-43
	*Oryza*	*meridionalis*	77.3	2056	88.0	1.14	1.54E-36
	*Oryza*	*barthii*	80.4	2089	89.9	1.12	1.34E-33
	*Zea*	*mays*	83.6	2281	92.0	1.10	7.73E-33
	*Oryza*	*punctata*	75.2	1994	84.0	1.12	4.55E-22

*a, percentage of non-constitutive exons in the transcriptome/genome of each species; b, total number of REPAG 3′ SS; c, percentages of non-constitutive exons in the immediate downstream of b; d, fold enrichment of c over a.*

We then examined the functional clustering of the host genes of representative metazoan and plant species using DAVID functional clustering analysis (Huang et al., 2009). The clusters have common as well as highly specific ones among these diverse species (Table 2). For example, the nucleotide- or ATP-binding cluster is found in *C. briggasae*, *Gorilla gallus*, *Homo sapiens* and *Pan troglodytes*, and the cluster calcium in drosophila, rat and plant (*A. thaliana*), while as the cluster plastid is found in *A. thaliana* only. The clustered functional proteins range from membrane receptors, cytosolic signaling kinases, cytoskeleton/transport proteins, Golgi complexes as well as nuclear DNA/RNA binding proteins (Figures 3C–F). Non-protein-coding RNA transcripts are also found (Figure 3G). The resulting splice variants change the protein sequences or non-protein coding RNAs. Therefore, the REPAGs are associated with diverse common as well as specific functions among different species across the animal and plant kingdoms.

**Table 2:** Most significantly clustered functions of genes containing the REPAG 3′ SS and downstream alternative exons in representative species using DAVID functional clustering analysis.

Division	Genus	Species	REPAG	Cluster name	Gene number in cluster	*p*-Value	Total number of mapped gene IDs
Metazoa	*Caenorhabditis*	*briggsae*	105	Nucleotide binding	9	1.30E-03	38
	*Danio*	*rerio*	716	Src homology-3 domain	17	1.90E-04	459
				Collagen	8	3.30E-04	
				Actin-binding	10	7.60E-04	
				Serine-threonine/tyrosine-protein kinase catalytic domain	11	2.80E-03	
				Pleckstrin homology domain	16	2.90E-03	
	*Drosophila*	*melanogaster*	130	Calcium	5	5.90E-03	99
	*Gallus*	*gallus*	1463	AAA+ ATPase domain	9	5.10E-03	379
				ABC transporter, conserved site	5	8.20E-03	
	*Gorilla*	*gorilla*	2193	ATP-binding	30	1.90E-04	1040
				MHC classes I/II-like antigen recognition protein	7	4.90E-04	
				Integrin-mediated signaling pathway	8	5.90E-04	
				Epidermal growth factor-like domain	13	7.00E-04	
				Protein kinase, catalytic domain	22	1.80E-03	
	*Homo*	*sapiens*	2281	ATP-binding	146	6.20E-11	1352
				Pleckstrin homology-like domain	49	9.80E-05	
				Natural killer cell lectin-like receptor binding	6	1.20E-04	
				Motor protein	21	1.90E-04	
				Very long-chain fatty acid-CoA ligase activity	6	2.30E-04	
	*Pan*	*troglodytes*	2518	ATP binding	128	2.60E-10	1103
				Pleckstrin homology-like domain	46	1.10E-05	
				Kinase	64	2.50E-05	
				Aorta development	7	2.20E-04	
				Src homology-3 domain	24	1.30E-03	
	*Rattus*	*norvegicus*	742	Microtubule motor activity	10	2.00E-05	487
				C2 calcium-dependent membrane targeting	14	9.40E-05	
				Dynein heavy chain domain	5	1.30E-04	
				Pleckstrin homology-like domain	23	3.20E-04	
				Immunoglobulin subtype 2	16	3.40E-04	
	*Xenopus*	*tropicalis*	656	Activation of GTPase activity	6	1.20E-04	217
				Regulation of vesicle fusion	5	7.70E-04	
				Endomembrane system	6	1.10E-03	
				Intracellular protein transport	9	2.10E-03	
				WD40 repeat, conserved site	8	9.30E-03	
	*Arabidopsis*	*thaliana*	1307	Transit peptide	97	1.80E-06	913
				Proteolysis involved in cellular protein catabolic process	14	3.70E-05	
Plants				Binding site: Substrate	20	2.30E-04	
				RNA-binding	35	4.70E-04	
				Metal ion-binding site: Calcium	6	6.40E-04	
				Plastid	72	8.10E-04	
	*Glycine*	*max*	2558	Phosphorelay signal transduction system	14	3.00E-06	1285
				DNA/RNA helicase, DEAD/DEAH box type, N-terminal	16	2.50E-05	
				Cyanoamino acid metabolism	13	4.80E-05	
				Glycoside hydrolase family 3	7	1.20E-04	
				Adaptor protein complex AP-4, epsilon subunit	4	1.20E-04	
				Glycolysis/Gluconeogenesis	22	1.30E-04	

### Purine- Including G- Triplets Are Significantly Enriched in the Aberrantly Used 3′ SS of SF3B1- or U2AF35-Mutated Cancer Samples

The REPAG 3′ SS comprise 0.76% of all 3′ SS (or 24% of purine-rich 3′ SS) in the human genome. They are relatively weaker splice sites that contribute to splicing inhibition through the bound hnRNP H/F (Sohail et al., 2014; Sohail and Xie, 2015b). To assess the importance of such 3′ SS in cell function or diseases, we examined the enrichment level of GGG between the −15 and −3 positions, together with pyrimidine- and the other purine-triplets, in the aberrantly used 3′ SS of human cancer samples containing mutations of 3′ SS factors, specifically SF3B1 or U2AF35 (Brooks et al., 2014; Darman et al., 2015; Alsafadi et al., 2016; Agrawal et al., 2017; Kesarwani et al., 2017).

**FIGURE 4:**
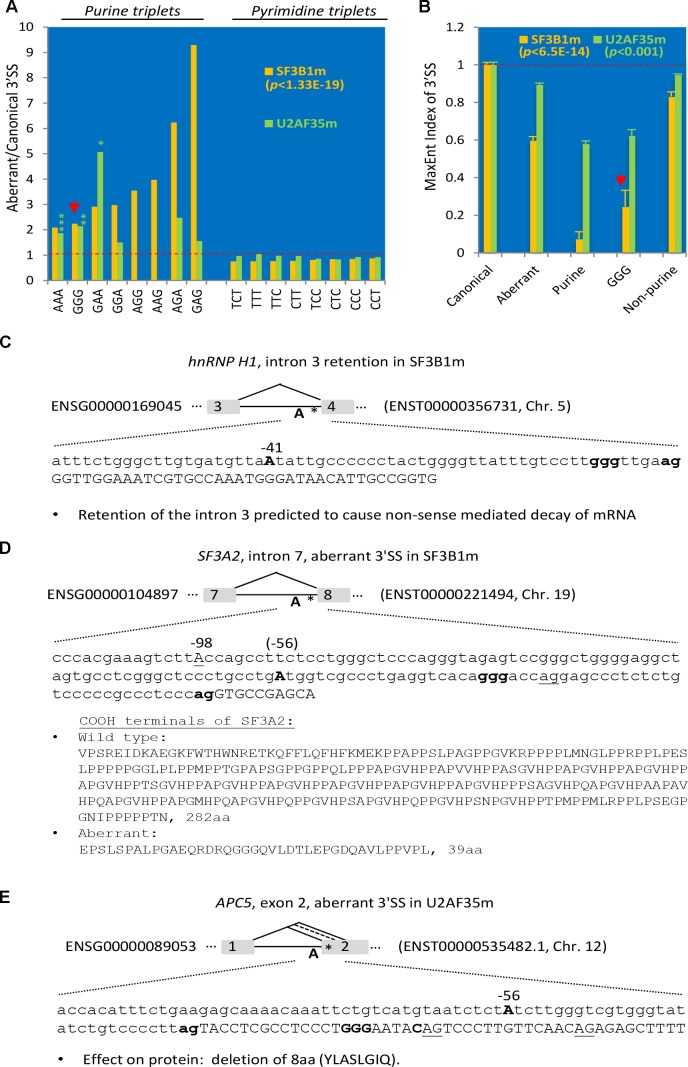
Enrichment of purine-triplets including GGG at the aberrantly used 3′ SS of SF3B1- or U2AF35-mutated human cancer samples. **(A)** Ratio of the percentages of pyrimidine (C/T) or purine (A/G) triplets between the –15 and –3 positions of aberrantly used over disrupted canonical 3′ SS (as baseline 1). *N* = 860 for SF3B1m (yellow) and 4,947 for U2AF35m (green) aberrant 3′ SS. For all triplets of SF3B1m, *p* < 1.33E-19. For U2AF35m, all pyrimidine triplets, *p* < 0.05; purine-triplets, ^∗^*p* < 0.05, ^∗∗^*p* < 0.01, ^∗∗∗^*p* < 0.001; AGG/AAG: value not available due to no such triplets within the disrupted canonical 3′ SS; all others not significant. **(B)** Measurement of the 3′ SS strength by the MaxEnt levels of the aberrant relative to the disrupted canonical 3′ SS (as baseline 1) with purine-, G- or non-purine-triplets in the mutant samples. The highly significant *p*-values for all of the SF3B1m and U2AF35m samples in comparison with the disrupted canonical 3′ SS are indicated. **(C–E)** Three examples of aberrantly used 3′ SS in the SF3B1- or U2AF35-mutated cancer samples with possible effects on the mRNA transcript and/or proteins. The branchpoint A, GGG and disrupted canonical 3′ AG are in bold, and aberrant 3′ AG underlined. ^∗^location of GGG in the intron.

In the SF3B1-mutated (SF3B1m) cancer patient samples including chronic lymphocytic leukemia, breast cancer, and melanoma, the mutations are between Y623 and G742 (Darman et al., 2015), within the HEAT domain, which is required for interaction with the ATPase Prp5 and branch site selection (Tang et al., 2016). Among the 860 aberrantly used unique 3′ SS of these samples (Darman et al., 2015), we identified 41 GGG-harboring 3′ SS, significantly more abundant over that in the disrupted canonical 3′ SS (∼2.2 folds, *p* < 1.33E-19, Figure 4A, and Supplementary Table S5a). So are the relative abundance of all the other purine-triplets (ranging from 2.1 to 9.3 folds, *p* < 1.0E-30), even including those containing the “AG” dinucleotides, which are often absent between the −15 and −3 positions, part of the AG-exclusion zone at canonical 3′ SS (Gooding et al., 2006). In contrast, all of the pyrimidine-triplets are significantly less abundant within this region of 3′ SS.

In the U2AF35m samples of lung carcinoma or acute myeloid leukemia, the mutation is S34F/Y (Brooks et al., 2014), within the N-terminal zinc finger domain that binds RNA (Yoshida et al., 2015). In these mutants, we identified 127 GGG-harboring 3′ SS between the −15 and −3 positions of 4,947 aberrantly used unique 3′ SS (Supplementary Table S5b), significantly (*p* < 0.01) more abundant than that in the disrupted canonical 3′ SS. So are the AAA and GAA triplets; the others are either not significantly different from the canonical ones (GGA, AGA, GAG) or absent from the canonical 3′ SS (AGG, AAG). The pyrimidine triplets are either more or less abundant than that in the canonical 3′ SS but overall not as significantly different as that in the SF3B1 mutants.

The enrichment of the purine-/GGG- and reduction of pyrimidine-triplets between the −15 and −3 positions of 3′ SS in the SF3B1 and U2AF35 mutants likely weakens the splice site strength as these positions are often occupied by pyrimidine tracts at constitutive 3′ SS. We thus measured their strength by the relative levels of MaxEnt scores (Yeo and Burge, 2004). The aberrant 3′ SS, particularly the purine- and/or GGG-harboring ones, are significantly lower compared to the canonical or aberrant non-purine-triplet ones (Figure 4B). The aberrantly used 3′ SS of SF3B1 mutants are further lower than those in the U2AF35 mutants in general, consistent with their more significant enrichment of the purine-triplets. Therefore, the GGG- and the other purine-triplets likely weaken the cryptic 3′ SS in general, likely to prevent their usage in wild type conditions but this weakness could be overcome upon mutation of the 3′ SS factors.

Three examples of such aberrant splicing of splicing factors or cell cycle regulators related to the G tract or 3′ SS control are in Figures 4C–E: the *hnRNP H1* exon 4, *SF3A2* exon 8 and the *APC5* exon 2. In *hnRNP H1*, the intron 3 was retained in *SF3B1m*, resulting in early termination of the ORF and likely nonsense-mediated decay of the transcript. In *SF3A2*, a 30nt-upstream AG with a REPAG in the intron was used to replace the wild type 3′AG in *SF3B1m*, causing a 28nt insertion and disruption of the open reading frame. The resulting protein is replaced of 282aa containing a PAT1 superfamily domain (Topoisomerase II-associated protein PAT1) by a shorter 39aa peptide. In the *APC5*, a REPAG aberrant 3′ SS is used in U2AF35m samples, replacing the two flanking alternative 3′ SS, resulting in a 24nt in-frame deletion of 8aa peptide YLASLGIQ. Therefore, aberrant usage of the REPAG 3′ SS in the *SF3B1* or *U2AF35* mutants affect the splicing factors that target the GGG, other BP factors and cell cycle control genes.

Together, their enrichment in the 3′ SS factor mutants suggests that the REPA and REPAGs are part of the weak cryptic 3′ SS that are prone to be aberrantly used in these human cancers.

### The REPAGs Are Associated With More Distant Branch Point Motifs

**FIGURE 5:**
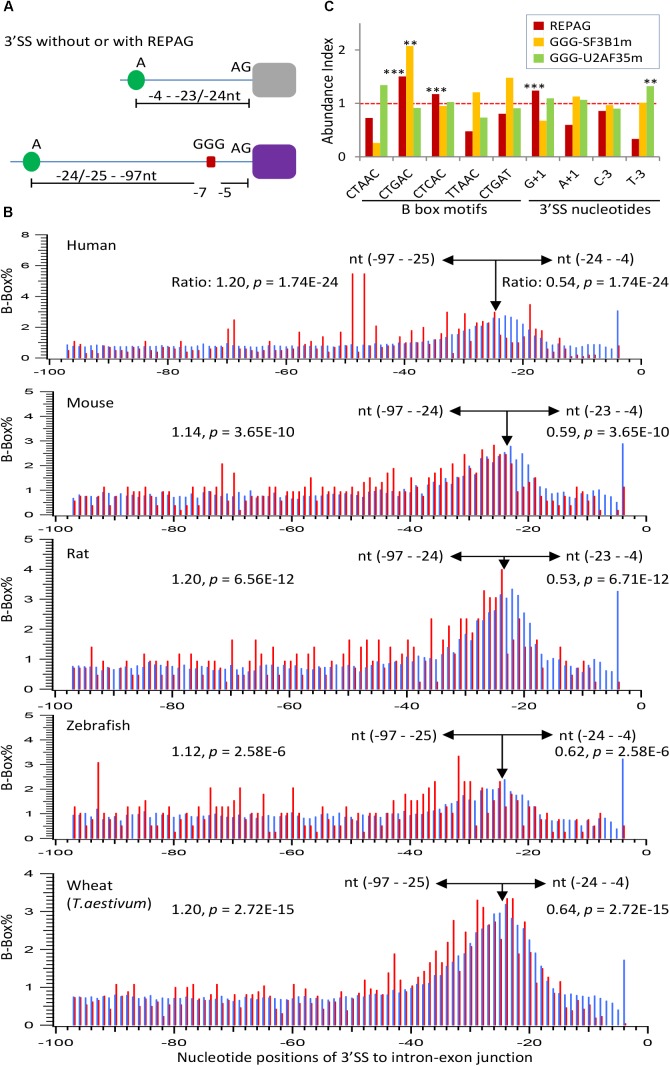
Properties of the REPAG-associated branchpoints. **(A)** Diagram of the major changes of the positions of branchpoint motifs in REPAG 3′ SS. **(B)** Percent distribution of the branchpoint motifs in the REPAG 3′ SS (red) and the control 3′ SS or the whole genome (blue), showing reduction of branchpoint motifs (B-boxes) from the –24 to –4, and exclusion from the –7 to –5 regions in the REPAG 3′ SS, accompanying their increase in the farther upstream regions from the 3′ AG. **(C)** Over-represented motifs of the five B-boxes or nucleotides (–3 or +1 positions) in the last 100nt of REPAG 3′ ends (red, *n* = 2,281 3′ SS) or at the 3′ ends (with GGG between –15 and –3) in the SF3B1 (yellow, *n* = 60 3′ SS) or U2AF35 (green, *n* = 127 3′ SS) mutants, compared to those in the 270,591 unique 3′ SS of the whole human genome. Their abundance in the genome is taken as 1.0. ^∗∗^*p* < 0.01, ^∗∗∗^*p* < 0.001. All of the samples without stars are not significantly different from that in the genome.

We have shown previously that the REPAGs are between the Py and 3′ AG to weaken the 3′ SS by their bound hnRNP H/F (Sohail and Xie, 2015b). However, their relationship to the corresponding BPs remains unclear. A large group of the human BPs and consensus pentamer motifs (B-boxes) have been identified by sequencing the lariats of human transcripts (Mercer et al., 2015). A number of the consensus motifs are predicted to have high affinity with U2 snRNA and overlap well with experimentally verified ones (Mercer et al., 2015). These predicted B-boxes exhibit 2.1 fold conservation over surrounding sequence in 100 vertebrates; several of them have similar frequency distribution at the 3′ SS from zebrafish to humans (Mercer et al., 2015). We thus examined the position distribution of the top 5 motifs (CTAAC, CTGAC, CTCAC, TTAAC, and CTGAT) in terms of affinity to U2 snRNA and abundance in the human genome, within the last 100nt of introns of human and vertebrate model organisms mouse, rat and zebrafish, as well as a plant species (common wheat). The peak positions of the motif’s Adenosine are all between −24 and −22 (Figures 5A–B), suggesting that the enrichment peaks of the B-boxes over the surrounding sequences is conserved in all these species including plants. These positions are also consistent with the experimentally verified 90% of human BP regions between −19 and −37 with a median of −25 (Mercer et al., 2015). Interestingly, the REPAG 3′ SS contain significantly less BP-Adenosine close to the 3′AG (between −24 and −4) than the control 3′ SS or genome background in each species (Figure 5B), in particular the BP-Adenosines are excluded between the −7 and −5 positions. This is accompanied by a significant increase between the −25/−24 and −97 positions (Figure 5B). Such distant BP motifs can be seen in the examples in Figures 3C–G: the positions of their potential BP motif-As range from −53 to −117 nt upstream in the introns. In humans, the BP-A peaks at −47 and −49 positions with significantly higher occurrences of alternative exons (100%, *n* = 110, compared to 97%, *n* = 140, between −24 and −4 positions, *p* < 0.005). An increase of alternative exons was also seen for the zebrafish 3′ SS with BP-As between −97 and −25 (88%, *n* = 260) in comparison with that between −24 and −4 (78%, *n* = 32, *p* < 0.05) but not in mouse and rat species. Therefore, the REPAGs are significantly associated with reduced BP motifs near the 3′ AG and more distant BPs across the animal and plant kingdoms, and may contribute to alternative splicing in some species.

Analysis of the five B-box motifs in the human REPAG 3′ SS showed that the CTGAC motif is most significantly enriched over the genome background (*p* = 8E-18, Figure 5C), similar to that in the aberrant 3′ SS in the SF3B1-mutated melanoma (Alsafadi et al., 2016). CTCAC is also enriched, to a lesser extent. The other three motifs are significantly under-represented (*p* ≤ 8.5E-5). In addition, the G_+1_ nucleotide is also enriched in these 3′ SS (*p* = 1.6E-27). Interestingly, of the 60 aberrantly used, GGG-harboring 3′ SS of SF3B1 mutant samples (Supplementary Table S4a), the abundance of the CTGAC motif is also significantly higher than in the genome background (Figure 5C). In contrast, the abundance of the other motifs or 3′ SS nucleotides in these samples are not significantly different from the background, and none of these motifs were significantly enriched in such 3′ SS of U2AF35 mutants. However, the latter are enriched of the T_−3_ (*p* < 0.01). Therefore, the REPAG 3′ SS or aberrantly used GGG-harboring 3′ SS are preferentially associated with certain 3′ SS sequences, particularly the branchpoint motif CTGAC.

### The REPA G Tracts Evolved in a Species- or Phylum-Specific Way

**FIGURE 6:**
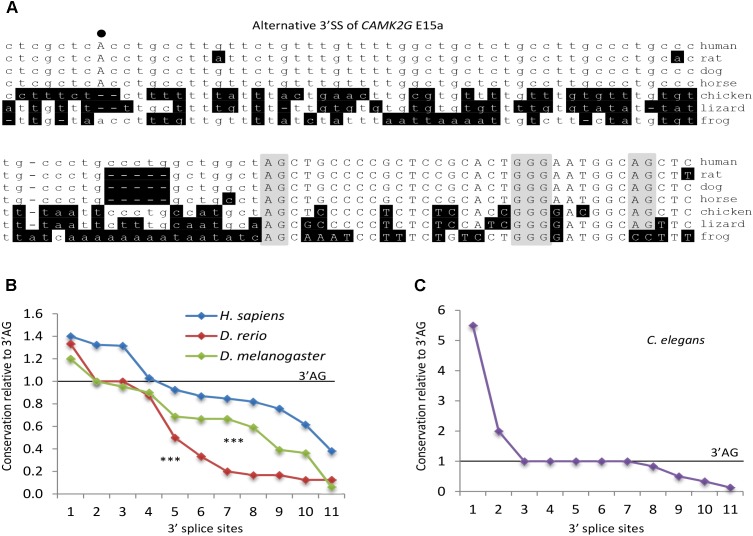
Conservation level of REPAGs of different species. **(A)** An example of the evolutionary conservation of the REPAG and corresponding 3′ SS motifs, by Clustal W alignment of the 3′ SS of the *CAMK2G* exon 15a (Figure 3C). Note that the GGG and upstream 3′ AG are conserved in all these species but the potential branchpoint A, its B-box motif and downstream 3′ AG are not. Nucleotides different from the human gene are shaded in black. The 3′ AGs and GGG are shaded in gray, and the potential BP A in bold and uppercase. (**B–C)** Conservation level of REPAGs relative to the corresponding 3′ AGs in different species. Frequencies of ten randomly chosen REPAGs and those in Figures 3C–G of each species were normalized to the frequencies of the corresponding 3′ AG (level = 1.0) among the species in their UCSC Multiz groups (100 vertebrates for human, 8 vertebrates for zebrafish, 25 insects for drosophila and 26 nematodes for *C. elegans*), and ranked from high to low for each species in the graph. ^∗∗∗^*p* < 0.001 for both drosophila and zebrafish in pairwise comparison with the human REPAGs. The *C. elegans* REPAGs are distinct from the others in their conservation levels; therefore they are displayed in a separate graph. The motif frequencies are listed in Supplementary Table S6.

In our previous studies (Sohail et al., 2014; Xie, 2014; Sohail and Xie, 2015b), most of the human REPAGs can be found with the 3′ SS in mammals but not the other vertebrates. To estimate the conservation level of the REPAGs here from diverse species, we examined the frequency of those from Figures 3C–F plus ten more randomly chosen from each species using the alignment information from the UCSC Multiz (Blanchette et al., 2004; Karolchik et al., 2014). Their frequencies vary widely: higher, same or lower than the corresponding 3′ AG (Figure 6 and Supplementary Table S6). More than half of those from human, zebrafish and fly are less conserved than the 3′ AG; in particular 82% of the zebrafish ones are specific for this species only (Figure 6B). Overall, human REPAGs are relatively more conserved than those in zebrafish or fly. For the *C. elegans* REPAGs, the conservation levels relative to the 3′ AG are more complex (Figure 6C and Supplementary Table S6). Five REPAGs are present with the 3′ AG in the same species (4 in *C. elegans* only and the other one also in 6 other species). The other six REPAGs and their 3′ AGs appear to have both undergone changes among the 26 nematode species leading to much higher or lower conservation levels of the REPAGs relative to the 3′ AGs. Therefore, relative to the 3′ AG, the REPAGs could be either more or less conserved but the extent varies depending on the species, suggesting that both have undergone dynamic changes in a species- or phylum-specific way during evolution, particularly in *C elegans* and the other nematodes. The changes likely contribute to species-specific alternative splicing by adding to the repertoire of regulatory *cis*-acting elements.

## Discussion

In this study, we have extended our previous finding about the REPAGs from mainly humans to more than a thousand species across the eukaryotic kingdoms. This has identified several new features of these elements. They are: (1) widely distributed across kingdoms, (2) highly associated with alternative splicing in more than 100 species of mainly vertebrates and plants, of genes with diverse functions, (3) highly enriched in human cancers with 3′ SS factor mutations, (4) associated with more distant branchpoints particularly the CTGAC motif, and (5) evolved in a species- or phylum-specific way. These results together with our previous findings provide an overview of the elements in eukaryotes, their role in alternative splicing and the diversification of gene transcripts in cell function and cancer.

### Enrichment of REPA G Tracts Diversifies Gene Products in Metazoa and Plants Through Alternative Splicing

The level of alternative splicing is higher in metazoa than that in fungi and protists (Barbosa-Morais et al., 2012; Merkin et al., 2012; Nguyen et al., 2018). Some plants also have relatively high levels (e.g., 40% of genes in maize *Zea mays* and 42% in *A. thaliana*) (Filichkin et al., 2010; Thatcher et al., 2014). These high levels of alternative splicing require corresponding control RNA elements for proper splicing. There are 244,674 REPAGs in the metazoan and plant genomes (0.8% of all 3′ SS, Supplementary Table S3, 1,064 ± 941, *n* = 230 species/lineages). Such widespread distribution together with their splicing inhibition effect suggests substantial contribution of these regulatory elements to the generation of splice variants in metazoa and plants.

We have shown that in humans these REPA G tract-containing genes are most significantly involved in cancer-related functions (Sohail and Xie, 2015b). Here their widespread presence among different species goes far beyond cancer, to associate with a wide variety of gene functions including cell’s response to external environment (e.g., signaling, movement), as well as core DNA/RNA/glucose metabolism or highly specific plastid functions in plants (Table 2).

In a step further from their association with cancer, we show that they are highly enriched in the aberrant 3′ SS in cancers containing U2AF35 or SF3B1 mutations (Figure 4). Interestingly, the aberrant intron retention of hnRNP H1 is anticipated to cause NMD and reduced expression of the hnRNP H1 (Darman et al., 2015) (Figure 4C), which could augment the usage of cryptic splice sites. Also the SF3A2 aberrant 3′ SS usage results in protein truncation of a conserved domain, likely to weaken the U2 snRNP function as well, again increasing the usage of aberrant splice sites. Therefore, the SF3B1 mutation-induced splicing factor changes are expected to further enhance aberrant splicing in cancer patients. Together the REPA-G tracts represent a special class of RNA elements highly enriched in the aberrant splicing events in cancer.

### REPA G Tract Inhibition of Cryptic 3′ SS and Influence by Distant Branch Points

These REPA G tracts are splicing silencers that tend to make the host 3′ SS skipped (Sohail et al., 2014; Sohail and Xie, 2015b). What was surprising at first thought is the significant enrichment of these silencers at the aberrantly used 3′ SS in mutants of either SF3B1 or U2AF35 (Figure 4). One reasonable scenario is that in the wild type cells they are splicing silencers to weaken the cryptic 3′ SS, as the other purine-triplets (Figure 4A), to prevent their usage. However, in the mutants particularly the SF3B1m, the weakness of the cryptic 3′ SS could be overcome to different extents, for example by help from the G_+1_ and/or new BPs with better B-box sequences CT(G/A)AC (Figure 5C) for stronger U2 snRNA binding (Darman et al., 2015; Mercer et al., 2015; Alsafadi et al., 2016). The CTGAC motif enrichment also occurs in the REPAG 3′ SS in the genome (Figure 5C), where it likely compensates the silencer effect of REPAG for the splice site to be alternatively used. The different extent of enrichment/usage of such 3′ SS perhaps reflects the different sensitivity of the SF3B1 or U2AF35 mutants to the strength of the cryptic splice sites and the polypyrimidine tract in particular.

We have shown previously that the REPAGs inhibit 3′ SS usage by its *trans*-acting hnRNP H/F to interfere with U2AF65 binding (Sohail et al., 2014; Sohail and Xie, 2015b), which is in a tight heterodimer with U2AF35 (Zamore and Green, 1989); thereby preventing their interaction with the Py and 3′ AG, respectively (Sohail and Xie, 2015a,b). Here they are also associated with more distant BPs from the 3′ AG (Figure 5). Even these are within 100nt in the intron end, not as distant as those found in other cases (Gooding et al., 2006), they are still enriched of alternative exons in some species, consistent with a previous genome-wide observation on the effect of distant BPs (Corvelo et al., 2010). Therefore, these GGG motifs likely regulate splicing with contribution from the more distant BPs as well.

Another question is what the functions are of such G tracts at the 3′ SS of fungi and protists while they are not as enriched over the 3′ SS and genome background as those in metazoa and plants. One possibility is that the corresponding 3′ SS factors have evolved to be functionally compatible with the G tracts, as the 5′ SS U1 snRNA or the LS2 protein in other cases (Taliaferro et al., 2011; Nguyen et al., 2018), so that they are not as inhibitory of splicing as in humans (Sohail et al., 2014; Sohail and Xie, 2015b). Or they perhaps have other functions in RNA metabolism that remains to be identified.

G-quadruplexes close to the 5′ splice site have been reported to promote alternative splicing of the upstream exon (Huang et al., 2017). However, they require at least four spaced repeats of GG, which are not present in the 2,281 human REPAGs (Supplementary Data Files S1, S2). Besides, the REPAGs are 3′ SS splicing silencers instead of enhancers. Therefore, the REPAGs apparently act distinctively on splicing from the quadruplex model.

In summary, the widespread separation of the Py and 3′ AG by the REPAGs across kingdoms and their association with alternative exons indicate that these independently evolved regulatory elements and this unique class of introns contribute greatly to the transcriptome and proteome diversity through alternative splicing.

## Materials and Methods

### Genome Data

The GenBank-format files of the genomes of all species examined here were downloaded from the release 91 (mostly vertebrates) or Genome release 38 (invertebrates and others) of the Ensembl databases, of which the transcripts are based on experimental evidence (Aken et al., 2016). The REPA search is based on our published script and criteria (Sohail et al., 2014). Briefly, 3′ SS containing more than 4 purine nucleotides between the −10 and −3 positions at the AG intron end were output from the annotated genomes. Of these, 3′ SS containing GGG between the −15 and −3 positions were output as REPAG 3′ SS. This search parameter has given most of the 3′ SS as authentic REPAG 3′ SS, for example, about 93% in humans. Besides the extracted data in the figures, the full species lists of such 3′ SS nucleotide matrices are in Supplementary Tables S1, S2, G tract and alternative splicing enrichment in Supplementary Tables S3, S4, respectively, and the individual 3′ SS sequences of all of the species in Supplementary Data Files S1, S2. The species conservation of REPAGs and 3′ SS motifs were examined in the Multiz-alignment sequences of the UCSC Genome Browser (Blanchette et al., 2004; Karolchik et al., 2014).

### Analysis of Constitutive vs. Non-constitutive Exons

We compared the paired coordinates of the genome nucleotide positions of all the annotated exons of genes with more than one transcript in the Ensembl databases. Exons with their both coordinates of a pair present in all of the annotated transcripts are considered constitutive exons, or else, non-constitutive or alternative exons. The criteria were applied to estimate the enrichment of transcriptome-wide alternative splicing of exons immediately downstream of REPAG 3′ SS among the species or among groups of 3′ SS with different branchpoint positions. The Python script used is in Supplementary Data File S3.

### Aberrant 3′ SS in Cancer Patients or Cell Lines

Upstream 3′ splice sites of exons/genes in SF3B1 mutants of the HEAT domain or U2AF35 S34F/Y mutants were obtained from the deposited sequences or exon coordinates published by Darman et al. (2015), or by Brooks et al. (2014). Here the extracted REPAGs-harboring unique 3′ SS of individual exons/genes are listed in Supplementary Tables S5a,b.

### Statistical Analysis

Hypergeometric test was used in the analysis of the density of REPAG motifs and non-constitutive exons or positions of BPs. Student’s paired *t*-test was used for the comparison of MaxEnt scores for the 3′ SS and the conservation levels of REPAGs of different species.

## Author’s Note

The manuscript has been deposited as a preprint at BioRxiv https://www.biorxiv.org/content/early/2018/07/06/363804.

## Author Contributions

HN performed computer analysis of raw data. JX analyzed the data and wrote the manuscript.

## Conflict of Interest Statement

The authors declare that the research was conducted in the absence of any commercial or financial relationships that could be construed as a potential conflict of interest.

## Supplementary Material

TABLE S1 Nucleotide matrices of purine-rich 3′ SS of 1175 species/lineages.

TABLE S2 Nucleotide matrices of REPAG 3′ SS of 1031 species/lineages.

TABLE S3 Percentages of purine-rich, REPAG 3′ SS and corresponding G% of each of the 1031 species/lineages.

TABLE S4 Enrichment of alternative exons downstream of REPAG 3′ SS in different species.

TABLE S5 GGG-harboring 3′ SS between the −15 and −3 positions in cancer samples or cell lines. **(A)** in SF3B1 mutant samples. **(B)** in U2AF35 mutants.

TABLE S6 Conservation levels of REPAG and corresponding 3′AG of 11 individual 3′SS from the several species in Figures 6B,C.

DATA FILE S1 Sequences of REPAG 3′ SS from each species of fungi.

DATA FILE S2 Sequences of REPAG 3′ SS from each species of metazoa, plants and protists.

DATA FILE S3 Python script to search for non-constitutive exons from the Ensembl-annotated transcriptomes/genomes.
