# Supplementary material for: Widespread Separation of the Polypyrimidine Tract From 3′ AG by G Tracts in Association With Alternative Exons in Metazoa and Plants
Source: Front Genet. 2019 Jan 14;9:741. doi: 10.3389/fgene.2018.00741 (PMC6339879; doi:10.3389/fgene.2018.00741)
Supplement: DATA FILE S3 — Python script to search for non-constitutive exons from the Ensembl-annotated transcriptomes/genomes. [file Data_Sheet_3.pdf]

```
#!/usr/bin/env python
# -*- coding: utf-8 -*-
"""
Created on Wed Sep 28 15:01:15 2016

Script will output constitutive exon for more than 1000 species
- Output path can be changes in line code: save_path =
'/home/xielab101/Documents/Species_Sequences/Release_38_91'
- Input path which contains directory of species, structures of folder as an
example below:
    1000_Species/Data_Genome_R36_90/ | Fungi_Release36
                                     | Metazoa_Release36
                                     | Metazoa_Release90
                                     | Plants_Release36
                                     | Protists_Release36
    Input path can be changed in main function in line code:
base_path='/media/xielab101/Seagate_Backup_Plus02/1000_Species/Data_Genome_R36_9
0/'
- Before run this script, download genbank file of all species and place them in
folder with above structure, downloaded files from ftp server are in zip format,
use command below to extract all files
  find /media/xielab101/Seagate_Backup_Plus02/1000_Species/Data_Genome_R36_90/ -
name "*.gz" -exec gunzip {} \;
@author: xielab
"""
import os
from pyrnaseq_util import find_matched_files
from Bio import SeqIO
save_path = '/media/xielab101/URMI/human_constitutive_exon_Rel93'

def extract_constitutive_exon(f,constituve_exon_list):

    genebank_file = SeqIO.parse(f, "genbank")
    for gb_record in genebank_file:
        chromosome = gb_record.name
        RNA_count = 0
        exon_list=[]
        constitutive_exon=[]

        for gb_feature in gb_record.features:
            if gb_feature.type=='gene':
                if RNA_count > 1:
                    if len(constitutive_exon) > 0:
                        for exon in constitutive_exon:
                            constituve_exon_list.append(exon)
                RNA_count = 0
                exon_list=[]
                constitutive_exon=[]
                if 'gene' in gb_feature.qualifiers:
                    gene_name = gb_feature.qualifiers['gene'][0] # get gene name
                else:
                    gene_name = 'None'
            if gb_feature.type=='exon':
                if RNA_count > 1:
                   if len(constitutive_exon) > 0:
```

```
                        for exon in constitutive_exon:
                            constituve_exon_list.append(exon)
                RNA_count = 0
                exon_list=[]
                constitutive_exon=[]
                break
            if gb_feature.type=='mRNA' or gb_feature.type=='misc_RNA':
                RNA_count +=1
                exon_list=[]
                num_sub_feature=len(gb_feature.sub_features)
                strand= gb_feature.location.strand
                if num_sub_feature:
                    pos_list=[]
                    for sub_feature in gb_feature.sub_features:
                        sub_start=sub_feature.location.nofuzzy_start
                        sub_end=sub_feature.location.nofuzzy_end
                        pos_list.append(sub_start)
                        pos_list.append(sub_end)
                    for i in range(num_sub_feature):
                        start=pos_list.pop(0)
                        end=pos_list.pop(0)

exon_list.append([start,end,strand,chromosome,gene_name])
                        if RNA_count == 1: # get first list only to check exon
of first in other list or not

constitutive_exon.append([start,end,strand,chromosome,gene_name])
                else:

exon_list.append([gb_feature.location.start.position,gb_feature.location.end.pos
ition,strand,chromosome,gene_name])
                    if RNA_count == 1:

constitutive_exon.append([gb_feature.location.start.position,gb_feature.location
.end.position,strand,chromosome,gene_name])
                #print exon_list
            if RNA_count != 0:
                remove_exon_list=[]
                for exon in constitutive_exon:
                    if exon not in exon_list:
                       remove_exon_list.append(exon)
                for remove_exon in remove_exon_list:
                    constitutive_exon.remove(remove_exon)


def out_report(constituve_exon_list,dir_name,ensenbl_division):
    if len(constituve_exon_list) == 0:

        print "No Constitutive Exon Found in " + dir_name

    else:
      completeName = os.path.join(save_path, ensenbl_division + '_' + dir_name
+'.txt')
      out_file=open(completeName,'w')
```

```
      out_file.write('Exon_Start' + '\t' + 'Exon_End' + '\t'+ 'Strand' + '\t' +
'Chromosome' + '\t' + 'GeneID' +'\n')
      for item in constituve_exon_list:
         out_file.write(str(item[0]) + '\t' + str(item[1]) + '\t' + str(item[2])
+ '\t' + str(item[3]) + '\t' + str(item[4]) + '\n')
      out_file.close()

def main():

base_path='/media/xielab101/Seagate_Backup_Plus02/1000_Species/Data_Genome_R36_9
0/' # parent Folder contains files of spiceces
    list_dir = os.listdir(base_path)
    sorted_list_dir = sorted(list_dir)
    print sorted_list_dir
    for ensenbl_division in sorted_list_dir:
        base_path1=base_path+ensenbl_division+'/'
        print base_path1
        list_dir1 = os.listdir(base_path1)
        list_dir_sort = sorted(list_dir1)
        for dir_name in list_dir_sort:
            print 'Species: ' + dir_name
            full_directory = os.path.join(base_path1,dir_name)
            matched_files
=find_matched_files(full_directory,'.dat',file_format='',folder_ignored=['files'
])
            constituve_exon_list=[] # Store seq of each spieces
            for f in matched_files:
                print f
                extract_constitutive_exon(f,constituve_exon_list)
            print len(constituve_exon_list)
            out_report(constituve_exon_list,dir_name,ensenbl_division)

    print ('Done')

if __name__=='__main__':
    main()
```
